# Garden-based interventions and early childhood health: an umbrella review

**DOI:** 10.1186/s12966-020-01023-5

**Published:** 2020-09-22

**Authors:** Kara R. Skelton, Chenery Lowe, Daniel A. Zaltz, Sara E. Benjamin-Neelon

**Affiliations:** Department of Health, Behavior and Society, Johns Hopkins Bloomberg School of Public Health, 624 N. Broadway, Baltimore, MD 21205 USA

**Keywords:** Early childhood, Early years, Child nutrition, Gardens, Preschool, Physical activity, Agriculture

## Abstract

**Background:**

Garden-based interventions show promise for improving not only child nutrition, but other indicators of child health. Yet, existing systematic reviews of garden-based interventions often focus on one particular health outcome or setting, creating a need to holistically summarize review-level evidence on the role of garden-based interventions in early childhood. To fill this gap, we performed an umbrella review of garden-based interventions to examine their role in early childhood health promotion for children ages 6 years and younger, examining effective components of garden-based interventions and critically evaluating existing evidence.

**Methods:**

We searched the following databases: PubMed, PubMed, PsycINFO, ERIC, CINAHL, Embase, Scopus, OVID-Agricola, and CAB Direct, limiting to reviews published from 1990 to August 2019. Of the 9457 references identified, we included a total of 16 unique reviews for analysis.

**Results:**

Across reviews, garden based-interventions were most effective at improving nutrition-related outcomes for children, including nutritional status and fruit and vegetable consumption. Few reviews examined child health outcomes of garden-based interventions that were not nutrition related, such as physical activity, or academic performance. Across settings, there was the most evidence in support of garden-based interventions conducted in home gardens, compared to evidence from early care and education or community settings. We were unable to report on most effective components of garden-based interventions due to limitations of included reviews.

**Conclusions:**

Existing evidence is difficult to interpret due to methodological limitations at both the review and primary study level. Therefore, the lack of evidence for certain child health outcomes should not necessarily be interpreted as an absence of an effect of garden-based interventions for specific outcomes, but as a product of these limitations. Given the breadth of evidence for garden-based interventions to improve a number of dimensions of health with older children and adult populations, we highlight areas of future research to address evidence gaps identified in this umbrella review. Further research on the role of garden-based interventions, including their impact on non-nutrition early childhood health outcomes and how effectiveness differs by setting type is necessary to fully understand their role in early childhood health promotion.

**PROSPERO registration:**

CRD42019106848.

## Background

In recent years, evidence from on the linkage between early childhood behaviors, sustained quality of life, and adult heath has come from the fields of epigenetics, nutrition, physical activity, and neuropsychology [1, 2]. This has led global and national organizations to prioritize interventions focusing on early childhood health [3–6]. Epigenetic research exploring the developmental origins of health has found that early childhood nutrition, in particular, is a vital determinant of adult health [1]. Early childhood environmental exposures, including nutrition, influence the gut microbiome and brain development, which are critical in the maintenance of a healthy immune response [1] and proper physical and socioemotional development [7]. With both early childhood physical activity and nutrition influencing cardiometabolic health [8], establishing healthy habits in these behavioral areas in early life quintessential to long-term health promotion [9]. Ultimately, the large number of habits developed during the first years of life and the impact early childhood health has on future health, makes it an ideal time for health promotion [9–11].

Effective approaches for early childhood health include both macro-and micro-level interventions. On a macro-level, policies and environments can promote early childhood health. At a micro-level, innovative strategies, such as interventions that include multiple components such as experimental learning and education have shown positive health outcomes for young children. These types of interventions may prove key in health promotion during early childhood. Garden-based interventions, which typically include hands-on learning with fruits and vegetables, nutrition education about food origins and systems, and production of fresh produce, have been associated with improved child health outcomes [12–18].

Garden-based interventions have demonstrated improvements in nutrition-related indicators, such as child nutritional status and food security, fruit and vegetable consumption, and weight status [14, 16, 17, 19–22]. Additionally, garden-based interventions have been utilized as a form of therapy for specific disorders and diseases, including autism spectrum disorder [23] and childhood cancer [19]. There may be additional health benefits of garden-based interventions, such as socioemotional development or biological health measures. Indeed, improvements in biological measures for children, such as vitamin A status (serum retinol) and iron deficiency anemia have resulted from garden-based interventions [24, 25]. These hands-on interventions may also increase outdoor physical activity [26] and improve academic performance [27]. For older youth and adults, gardens have improved mental health, and may help reduce anxiety, stress, and anger [16, 28, 29].

To date, several reviews have examined the impact of garden-based interventions on children and reported positive effects for many child health outcomes [15, 19, 27, 30–33]. However, the impact of garden-based interventions during early childhood is difficult to collate as few reviews have assessed multiple child health outcomes in the same article. Most existing reviews focused on a singular outcome, such as fruit and vegetable intake [30], academic performance [27], or mental health [34]. Further, some reviews focused on a single type of gardening program (e.g., farm-to-preschool) [32], rather than exploring the multiple settings in which garden-based interventions can occur. Additionally, most reviews do not singularly focus on early childhood; rather, early childhood outcomes are included as sub analyses of the review. Thus, there is a need to comprehensively collate the evidence regarding the impact of garden-based interventions on a variety of early childhood outcomes. In effort to address this evidence gap, we conducted an umbrella review to summarize existing review level evidence of garden-based interventions on health outcomes for children ages 6 years and younger.

## Methods

For this umbrella review, we aimed to 1) identify and synthesize existing review-level evidence on garden-based interventions for children ages 6 and younger; 2) examine which components of garden-based interventions are most effective at improving child health outcomes; and 3) critically evaluate included reviews both narratively and quantitatively; and 4) identify potential gaps in the literature and highlight possible areas for improvement in the field of garden-based interventions, including but not limited to study design, measurement, and health outcomes. We used guidance from the Joanna Briggs Institute (JBI) Methodology for Umbrella Reviews [35] and the Cochrane Handbook’s Methodology for conducting an overview of reviews [36] to strategically create an a priori protocol for this umbrella review [37]. The published protocol for this review was developed in accordance with the Preferred Reporting Items for Systematic Reviews and Meta-Analyses Protocols (PRSIMA-P) 2015 Statement [38] and registered with PROSPERO (International Prospective Register of Systematic Reviews, CRD42019106848). We used the systematic review management software Covidence [39] to streamline the review process.

### Search strategy

In January 2019, we searched PubMed, PsycINFO, ERIC, CINAHL, Embase, Scopus, OVID-Agricola, and CAB Direct, restricting to articles published after January 1990. We also searched review registries, including the Cochrane Register of Systematic Reviews, the JBI Database of Systematic Reviews and Implementation Reports, and PROSPERO. We included the first 200 results of Google Scholar, when sorted by relevance. For included articles, we performed forward and backward citation searches to identify any relevant reviews. Prior to data analysis, we conducted an updated search for articles published between January and August 2019.

We crafted search terms using synonyms for gardening and young children used in prior reviews [15, 30, 31, 40], including additional terms created through collaboration with a medical librarian specializing in systematic reviews. For each database searched, we used database-specific controlled vocabulary and key terms. For databases without advanced search options (e.g., Google Scholar), we used a simpler search strategy that was comprised of a variation of gardening terms (e.g., “gardening”, “review”, and “children”). A pilot search informed the development of the final search strategy, which is located in Additional file 1.

### Eligibility criteria

We delineated a priori inclusion and exclusion criteria utilizing the population, intervention, context, outcome, and study design (PICOS) [41]. We applied eligibility criteria at both the systematic review and primary study level. For a review to be eligible for inclusion, at least one primary study had to meet all inclusion criteria. For example, if a review appeared eligible for inclusion, but further examination revealed no primary studies that met inclusion, we excluded the review.

#### Participants

We included reviews that included children ages 6 years and younger. A review did not have to include only children 6 years and younger; we included reviews with at least one primary study with our population of interest. We did not employ any limitations regarding gender, socioeconomic status, or specific child health conditions.

#### Intervention

We included systematic reviews that focused on or included garden-based interventions. As garden-based interventions are inherently complex to define due to variation in type and setting, we included any intervention that engaged children in active learning about nutrition, food systems, agriculture, or environmental health through connections with outside fruit or vegetable gardens or farms, raised garden beds, greenhouses, container gardens, microfarms, or other alternative gardening methods [37]. We also included farm-to-preschool and farm-to-child care programs, which often link young children with fresh produce from local farms.

#### Context

We included garden-based interventions occurring in any country and setting, including homes, early care and education programs (e.g., preschool or child care), community centers or community gardens, afterschool programs, and summer camps. We included garden-based interventions that focused on gardening interventions only, as well as multi-component interventions that included gardening.

#### Outcomes

We included reviews with at least one of the following child-level health outcomes of interest: nutrition-related behaviors (e.g., consumption, attitudes, preferences, dietary quality), nutritional status, anthropometric measures (e.g., body mass index (BMI), body fat percentile, BMI z-score), physical activity, cognition-related outcomes (e.g., academic performance, developmental milestones), mental health (e.g., social behavior, stress, anxiety), screen time, and biological outcomes (e.g., hemoglobin, serum retinol, microbiome). We excluded reviews that did not report on at least one of the child health outcomes of interest for our population of interest. We considered adverse or unintended consequences when noted in reviews. Although we included reviews that reported on both child and parent-level health outcomes, we extracted only child-level outcomes for our population of interest for analysis. We excluded reviews that included only parent-, school-, or community-related outcomes. We extracted health outcomes for our population of interest only. In instances that a review included multiple health outcomes of interest, but we could not disaggregate outcomes for our population of interest, we excluded that outcome.

#### Types of studies

We included peer-reviewed systematic reviews, with or without meta-analyses, published January 1990 through August 2019 [41]. We used the following definition of systematic review, which aligns with the definition of a systematic review provided in the PRISMA-P 2015 statement: a review which (a) has an explicit set of aims; (b) employs a reproducible methodology, including a systematic search strategy and selection of studies; and (c) systematically presents and synthesis characteristics of included studies [42]. We excluded reviews that failed to meet this definition. We included systematic reviews of studies that had randomized, quasi-randomized, and non-randomized designs. We excluded reviews that included qualitative studies only.

### Study screening and selection

We imported the associated Endnote X9 (Clarivate Analytics) library for each database search directly into Covidence. Citations were automatically de-duplicated as part of the import process. Two teams, each consisting of two reviewers, independently screened titles and abstracts. During this iterative screening process, Covidence automatically filtered citations into one of three lists, ‘Irrelevant”, “Resolve Conflicts”, and “Full Text Review”. We resolved disagreements between reviewers using consensus; no third reviewer was necessary. If a review team could not make an inclusion decision during the title and abstract screening phase, the article moved forward to full text review. After title and abstract screening, we gathered citations in their full-text, PDF form for full-text review. The same two teams of reviewers independently completed full-text screening, during which both reviewers had to agree on a final inclusion or exclusion decision.

### Data extraction

Three reviewers across two teams independently extracted data from included articles directly into a customized data extraction form within Covidence. For each included article, we extracted the following: citation details, aims or objectives, review type, eligibility criteria (e.g., population, setting, intervention type, study design), search strategy and results (e.g., number of databases searched, date range, inclusion of gray literature, number of included studies), relevant child-level health outcomes, and funding source. We also extracted data at the primary study level for eligible studies, which included citation details, population, setting, intervention type and design, results, limitations, and conclusions to enable us to account for primary study overlap [36]. We contacted corresponding authors for missing information and clarification, if needed.

### Quality appraisal

Three reviewers split into two teams independently performed quality assessment of included reviews via the AMSTAR 2 (A Measurement Tool to Assess Systematic Reviews) questionnaire [43]. The AMSTAR 2 is a 16-item validated quality assessment tool that allows for inclusion of both randomized and observational studies and as such, is not intended to be scored [44]. Reviewers resolved any discrepancies through discussion and consensus on appraisal criteria.

## Results

**Fig. 1:**
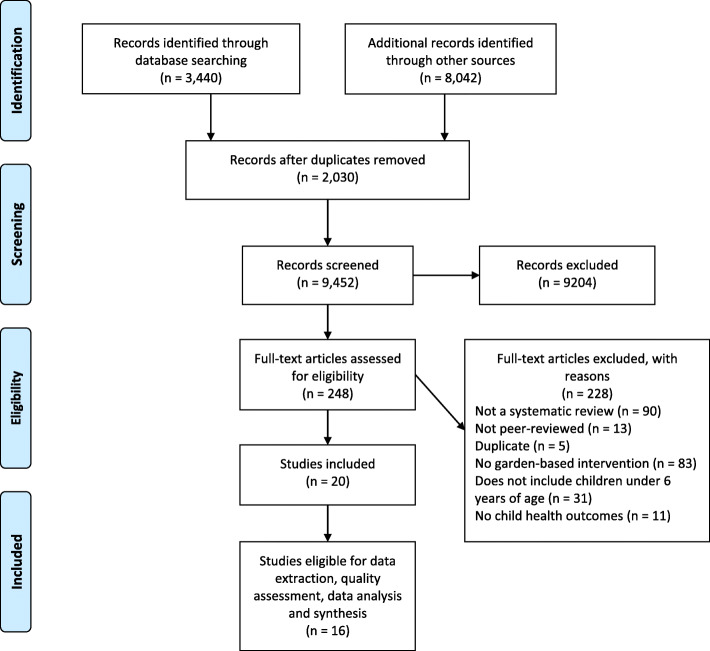
PRISMA flow diagram

Out of 9452 titles and abstracts screened for inclusion, 20 reviews were eligible. However, 4 reviews were previous versions of a living systematic review [45–48] and therefore, not included in data extraction. Fig. 1 describes results of the systematic search and study selection process, in accordance with PRISMA reporting guidelines [38]. See Additional file 2 for the full list of excluded studies.

### Description of included reviews

Table 1 details characteristics of the 16 included Systematic reviews [27, 30, 31, 48–60], including aim, topic area, interventions and populations included, databases searched, and funding source. Included reviews were published in English between 2004 and 2019. Five reviews included gray literature as part of their search strategy [31, 51, 52, 57, 59]. Five reviews focused on garden-based interventions and therefore, included only garden-based interventions [27, 30, 31, 54, 56]. Other reviews focused on improving nutrition status or healthy eating and included an array of agricultural, obesity prevention, nutrition education, and multi-component interventions. Similarly, some reviews examined one specific outcome (e.g., vegetable intake, physical activity) [49, 50], whereas others examined a number of outcomes (e.g., obesogenic behaviors) [60]. Three reviews included interventions that assessed vegetable-related outcomes only (e.g., intake, preferences, purchasing, provision) [49, 55, 59]; an additional 3 reviews included only interventions that measured fruit or vegetable outcomes [30, 48, 56]. Although only 5 reviews conducted formal meta-analysis [48, 50, 56–59], several reviews reported an inability to do so due to variation in study design and measures [27, 60], heterogeneity [55], and lack of sufficient data [58].

**Table 1:** Characteristics of included systematic reviews

Study	Aim	Topic Area	Intervention	Population	Databases Searched	Funding Source
Appleton *et al*. [49]	To systematically review the literature to identify all published interventions aiming to increase vegetable consumption	Vegetable intake	Any	Any	PubMed, PsycINFO, Medline & gray literature; up to Apr. 28, 2015	European Union’s Research and Innovation 7th Framework
Beets *et al.* [50]	To provide a systematic review of published research examining after-school programs targeting youth physical activity	Afterschool programs and physical activity	RCT or quasi-experimental	Children <18y	PubMed, ScienceDirect & EBSCOhost; 1980 to Feb. 2008	Not reported
Berezowitz *et al*. [27]	To collate findings on school garden interventions that include measures of academic performance and fruit and vegetable consumption	School gardens and academic performance	Any garden intervention lasting at least one month	K-12th graders in western cultures	CABI, Web of Knowledge, Web of Science, PubMed, Education Full Text, ERIC, and PsycINFO, up to May 2013	Centers for Disease Control & Prevention’s Community Transformation Grant
Berti *et al*. [51]	To critically review the literature concerning effectiveness of agriculture interventions in improving household nutrition status	Agriculture interventions and nutrition	Any agriculture intervention	No restrictions	Medline, Current Contents, Biosis Previews, PASCAL, AGRIS, and gray literature, 1985 to Nov. 2001	Canadian International Development Agency
Bhutta *et al*. [52]	To estimate the effects of undernutrition on childhood death and disability outcomes	Dietary diversification via agricultural production	Any agricultural intervention	Children and households in developing countries	PubMed, Current Contents, Helen Keller International, Medline, Biosis Previews, AGRIS, PASCAL & gray literature; 1995 to Nov. 2001	World Bank; International Centre for Diarrheal Disease Research, Bangladesh
Bird *et al*. [53]	To identify and evaluate the strength of evidence from interventions that assessed the impact of agricultural interventions on nutritional outcomes	Nutrition-sensitive agriculture	Quasi-experimental, RCT	Pakistan, India, Afghanistan, Nepal, or Bangladesh	Web of Science, Scopus, PubMed, CAB Abstracts, AGRIS, and gray literature; Jan. 2012 to Nov. 2017	Leveraging Agriculture for Nutrition in South Asia Research Consortium
Davis *et al*. [54]	To review school garden-based programs targeting dietary intake and related behaviors in children; to identify strategies and components employed by garden-based programs	School gardens and health outcomes	Any pre/post school garden intervention	Children of school age	Medline and Embase, and gray literature	No funding
Hendrie *et al*. [55]	To identify intervention characteristics and behavior change techniques associated with increasing children’s vegetable consumption	Vegetable intake	Any intervention evaluating effectiveness	Children 2-12y	PubMed, PsycINFO and CAB abstracts; 2004 to June 2014	Horticulture Innovation Australia Limited, CSIRO
Hodder *et al*. [48]	To assess the effectiveness, cost effectiveness and adverse events of interventions designed to increase the consumption of fruit or vegetables or both among children <5 years	Fruit and vegetable consumption	RCTs, cluster-RCTs, and cross-over trials	Children <5y	CENTRAL, MEDLINE, Embase, CINAHL, PsycINFO, ProQuest, WHO Clinical Trials Registry, Clinicaltrials.gov, Google Scholar; up to Aug. 25, 2019	Not reported
Langellotto *et al*. [56]	To examine the efficacy of garden programs for increasing children’s nutrition knowledge, fruit and vegetable preference, and fruit and vegetable consumption	Garden-based programs and child nutrition	Any nutrition education program	K-8th graders in the US	Google Scholar, PubMed, Web of Knowledge and *American Society of Horticultural Science* archives, and gray literature	Not reported
Masset *et al*. [57]	To inform policymakers on the effectiveness of interventions and suggest which designs, methods, and metrics should be used in future research	Agricultural interventions and nutritional status	Longitudinal project-controls and randomized field trials that aim to improve nutrition status	Children in low- or middle-income countries	Econlit, IBSS, PubMed, and Web of Science, AGRIS, & gray literature; 1990 to Sept. 2010	United Kingdom Department for International Development
Mikkelsen *et al*. [58]	To review published literature on healthy eating interventions in day care facilities and analyze effectiveness of different strategies in relation to their influence on children’s food choice	Healthy eating in preschools	Obesity prevention interventions, pre/post design or stronger	Children 3-6y	PubMed, Scopus, Web of Science & CINAHL; 1980 to 2014	Not reported
Nekitsing *et al*. [59]	To investigate effectiveness of interventions to increase young children’s vegetable intake through a comprehensive search that includes a variety of study designs and settings	Vegetable intake in young children	Any intervention aiming to increase child vegetable intake	Children 2–5y	Scopus, Medline, PsycINFO, CAB Abstracts, Embase, CINAHL, ERIC, CENTRAL, ProQuest, PubMed, & Web of Science, and gray literature; 2005 to Jan 2016	White Rose Social Sciences Economic and Social Research Council Collaborative Award
Ohly *et al*. [31]	To conduct a robust, mixed-methods systematic review of the health and well-being impacts of school gardening	Health and well-being impacts of school gardens	Any garden intervention in a school setting	Children <18y in OECD countries	MEDLINE, Embase, PsycINFO, HMIC, SPP, AEI, BEI, ASSIA, BNI, ERIC, AMED, CINAHL, and gray literature; up to May 2015	European Centre for Environment and Human Health
Savoie-Roskos *et al*. [30]	To identify effectiveness of gardening interventions to improve fruit and vegetable intake among children	Garden-based interventions and fruit and vegetable intake	Any community, school, or after-school garden intervention	Children 2-15y in developed countries	PubMed, Web of Science, CINAHL, Scopus; 2005 to Oct. 2015	No funding
Sisson *et al*. [60]	To determine effectiveness of obesogenic behavioral interventions in childcare centers across social ecological levels and describe strategies utilized	Obesogenic behavior interventions in child care	Any intervention targeting obesity, physical activity, or screen time	Children 3-5y in child care centers	PubMed, PsycINFO, Ovid; up to Jan. 2016	No funding

The level of scientific evidence presented in reviews varied, with some reviews finding no significant association between garden-based interventions and health outcomes, and other reviews reporting only positive outcomes. Across reviews, multi-component and multi-setting interventions appeared to be most effective [30, 48, 59, 60]. Of reviews that examined agricultural interventions, garden-based interventions [27, 30, 31, 54, 56] seemed to be more effective in improving nutrition-related indicators than other interventions, such as only nutrition education, agriculture technology, or livestock production.

Reviews included a total of 465 primary studies. The number of primary studies included in reviews that met our inclusion criteria ranged from 1 [50] to 6 [52] across reviews. Most reviews (*n* = 10) assessed the quality of original studies via an array of tools, including the Effective Public Health Practice Project Quality Assessment Tool [30, 31, 55, 59], Stetler’s Level of Quantitative Evidence [60], an adapted version of Critical Appraisal Skills Programme for RCTs [53], and the Cochrane Risk of Bias tool [48]. Two reviews developed their own rating system to appraise quality [51, 57] and one adapted the Cochrane Risk of Bias tool [58]. The remaining 6 reviews did not perform any quality assessment of original studies [27, 49, 50, 52, 54, 56].

### Quality of included reviews

Hodder et al. 2019 was the highest quality review, fulfilling 15 out of 16 of the AMSTAR 2 elements [48]. In contrast, Beets et al. fulfilled only one and partially fulfilled two AMSTAR 2 appraisal elements [50]. Most reviews included the PICO elements in their inclusion criteria, either explicitly or implicitly, whereas only 3 reviews explicitly stated they developed an a priori protocol [31, 48, 59]. A small number of reviews (*n* = 5) investigated publication bias and discussed its impact on review results [31, 48, 56, 59]. Of included reviews, Hodder et al. 2019 was the only review that reported on funding of included studies [48]; the remaining reviews did not report on funding for primary studies. Table 2 provides comprehensive results of the AMSTAR 2 quality appraisal.

**Table 2:** AMSTAR 2 appraisal

	PICO components	A priori Protocol	Study Design	Search Strategy	Study Selection	Data Extraction	Excluded Studies	Description of Included Studies	RoB Assessment	Reported Funding	Meta-analysis methods	Meta-analysis RoB	Discussion of RoB	Explanation of Heterogeneity	Publication Bias	Conflict of Interest
Appleton et al 2016 [49]	Partial Yes	No	No	No	Yes	No	Partial Yes	Partial Yes	No	No	N/A	N/A	Partial Yes	Partial Yes	Partial Yes	Yes
Beets et al 2009 [50]	Yes	No	No	No	No	No	No	Partial Yes	No	No	No	No	No	No	No	Partial Yes
Berezowitz et al 2015 [27]	No	No	No	Partial Yes	No	No	No	Partial Yes	No	No	N/A	N/A	Partial Yes	Partial Yes	No	No
Berti et al 2004 [51]	No	No	No	Partial Yes	No	No	No	Partial Yes	No	No	N/A	N/A	No	No	No	No
Bhutta et al 2008 [52]	No	No	No	Partial Yes	No	No	No	Partial Yes	Partial Yes	No	N/A	N/A	No	No	No	Yes
Bird et al 2019 [53]	Partial Yes	No	No	Partial Yes	Partial Yes	No	No	Partial Yes	Partial Yes	No	N/A	N/A	Partial Yes	No	No	Yes
Davis et al 2015 [54]	Partial Yes	No	No	No	No	No	No	No	No	No	N/A	N/A	No	No	No	Yes
Hendrie et al 2017 [55]	Yes	No	No	No	No	No	No	Yes	Partial Yes	No	N/A	N/A	Partial Yes	Partial Yes	No	Yes
Hodder et al 2018 [45]	Yes	Yes	Yes	Yes	Yes	Yes	Yes	Yes	Yes	Yes	Yes	Yes	Partial Yes	Yes	Yes	Yes
Langellotto et al 2012	Partial Yes	No	Yes	No	No	No	No	Partial Yes	No	No	No	No	No	No	Yes	No
Massett et al 2012	No	No	Yes	Partial Yes	Partial Yes	No	No	No	Partial Yes	No	Partial Yes	No	No	No	No	Yes
Mikkelsen et al 2014 [58]	Yes	No	No	Yes	No	Partial Yes	No	Yes	Partial Yes	No	N/A	N/A	No	No	No	Yes
Netkitsing et al 2018	Yes	Yes	Yes	Partial Yes	No	Partial Yes	No	Yes	Yes	No	Yes	Yes	Yes	Yes	Yes	Partial Yes
Ohly et al 2016 [31]	Yes	Yes	No	Yes	Yes	No	No	Yes	Yes	No	Yes	Yes	Partial Yes	Partial Yes	Yes	Yes
Savoie-Roskos 2017 [30]	Yes	No	No	No	No	No	No	Partial Yes	Yes	No	N/A	N/A	Yes	No	No	Yes
Sisson et al 2016 [60]	Yes	No	No	No	No	No	No	Partial Yes	No	No	N/A	N/A	No	No	No	Yes

### Overlap

We used a validated measure, the corrected covered area (CCA), to calculate the extent of overlap at the primary study level across included reviews [61]. We calculated the CCA by dividing the frequency of repeated occurrences of primary studies across reviews by the product of index publications and reviews, reduced by the total number of primary studies. The CCA was estimated to be 4.7, representing only slight overlap amongst included reviews [61]. The citation matrix used for calculating overlap is available in Additional file 3. Namenek Brouwer et al. was the article included in the most number of reviews (*n* = 7) [14].

### Garden-based interventions

Table 3 describes characteristics of garden-based interventions, including setting, country, relevant findings, and conclusions. Across included reviews, 24 unique primary garden-based intervention studies met inclusion criteria [14, 24, 25, 62–82] and were published between 1991 [70] and 2017 [25]. Most garden-based interventions (*n* = 15) were implemented in the home [24, 25, 64, 67–71, 73–77, 82] and 8 were conducted in school, afterschool, or early care and education settings [14, 63, 65, 66, 78–81]. Only one community garden-based intervention included our age group of interest [62].

**Table 3:** Characteristics and findings of garden-based interventions among included reviews

Review	Primary Studies^**a**^	Setting	Countries Included	Relevant Garden-based Intervention Findings	Conclusions of Review
Appleton *et al*. [49]	2/77	Home, school	South Africa, US	Multicomponent interventions reported success at improving vegetable intake and associated outcomes (e.g., selection of vegetables). A multicomponent intervention that included home gardening significantly improved vitamin A status of 2–5y children via production of yellow and dark green leafy vegetables.	There were many barriers to increasing vegetable intake. Although successful interventions have been published, the true value of these, both in cost-efficiency, long-term benefits, and sustainability are yet to be determined.
Beets *et al.* [50]	1/11	After-school	US	Physical activity: [MA] Positive effect sizes (Hedge’s g: 0.44 [95% CI:0.28–0.60], 6 studies) Herman 2006 effect size: 0.70 (95%CI: 0.05–1.36)	Afterschool programs that include some component of physical activity, can be effective in improving outcomes in children.
Berezowitz *et al*. [27]	1/16	School	US	Fruit and vegetable consumption: 71% of studies measuring fruit and vegetable intake reported significant improvements. Academic outcomes: Of 4 studies measuring academic outcomes, 3 showed improvements in science achievement and only 1 showed improvements in math scores.	School-based garden interventions improved or maintained both fruit and vegetable consumption and academic performance. Schools should consider school gardens as a hands-on tool to enhance science learning and potentially improve long-term fruit and vegetable consumption.
Berti *et al*. [51]	5/30	Home	Bangladesh, Vietnam, Guatemala, Thailand, Philippines	Nutrition outcomes improved in 11/13 home garden interventions. Of 17 projects ranked high or mid, 9 were home garden programs that aimed to improve nutritional status. All 9 home garden projects included nutrition education, often with another public health intervention. Of home garden interventions, 16/19 indicators were better in the intervention group.	Home gardening projects were more successful than other intervention types, perhaps because they are easily adoptable and may strengthen human capital. Almost all home gardening projects incorporated gender considerations, which may have partly been responsible for the positive effect on child nutrition outcomes.
Bhutta *et al*. [52]	6/29	Home	China, Vietnam, Iran, Laos, South Africa, Thailand	Included studies were found to have a positive effect on agricultural production and dietary intake. The 4 studies evaluating impact on nutritional status found a positive effect.	Although some agricultural interventions are potentially promising and culturally relevant, there is not enough evidence to suggest that these dietary diversification strategies are effective in improving nutritional status or micronutrient indicators on a large scale.
Bird *et al*. [53]	2/6	Home	India, Nepal	Dietary quality: 7 interventions reported improved dietary quality and diversity. Anthropometry: 4 papers reported a lack of convincing evidence	There was not strong evidence that agricultural interventions impacted final measures of nutritional status, the potential of agricultural interventions to improve intermediate outcomes provides support for continued research in this area
Davis *et al*. [54]	2/13	School	US	Garden programs resulted in improved attitudes towards, willingness to taste, identification of and self-efficacy to prepare and cook fruits and vegetables Vegetable consumption: 6/10 programs examined dietary intake, 6 found increased vegetable intake; 4 showed no effect Vegetable preference: Almost all (7/8) that measured found increased preference	The present analysis showed clear and consistent effects of school garden programs on improved dietary behaviors, with half of the studies showing increases in vegetable intake. Transition from start-up to long term maintenance of garden initiatives is an area of further work to enhance sustainability and thus the duration of health effects.
Hendrie *et al*. [55]	2/22	Home and community	US	Vegetable consumption*:* 12/22 studies conducted in home or community settings were effective short-term, with mean short-term change 29% (about ¼ to ½ serving of vegetables); 6/10 were effective long-term (6 + months)	Interventions targeting vegetable intake in home or community settings are generally effective and may increase intake by around 30%. Intervention effectiveness was associated with number of settings targeted and frequency of contact, but not length of intervention.
Hodder *et al*. [48]	2/63	Child care centers, community	US	Fruit and vegetable consumption: Both trials reported a positive intervention effect. [MA] Multicomponent interventions vs. no intervention had a small effect (SMD 0.34, 95% CI 0.10 to 0.57; 9 trials, 3022 participants; moderate-quality evidence), equivalent to an increase of 0.36 cups of fruit and vegetables per day. Data were insufficient to assess long-term effects.	Child-feeding practice interventions may lead to, and multicomponent interventions probably lead to, small increases in children’s intake of fruit and vegetables in the short term (less than 12 months). However, the quality of evidence is low and effect sizes may be too small for clinical utility.
Langellotto *et al*. [56]	2/20	School, after-school	US	Vegetable consumption: [MA] Gardening: significant increase [E++ = 0.42, CI = 0.07 to 2.07, df = 3]; Control: no significant effect Nutrition knowledge: [MA] Gardening: No significant effect [E++ = 0.21, CI = −1.19 to 0.43, df = 2]; Control: significant increase [E++ = 0.23, CI = 0.04 to 1.02, df = 2]Fruit consumption: [MA] Gardening: significant increase [E++ = 0.08, CI = 0.02 to 0.12, df = 1]; Control: no significant effect Fruit preference: [MA] Gardening: no significant effect [E++ = −0.02, CI = –0.20 to 0.01, df = 3]; Control: no significant effect Vegetable preference: [MA] Gardening: significant increase [E++ = 0.10, CI = 0.01to 0.19, df = 1]; Control: no significant effect	Gardening interventions had more positive significant effects (including both pre- and post-test comparisons and comparison to control groups) than nutrition education interventions or control conditions. These types of programs should receive more funding for rigorous research, including federal funding.
Masset *et al*. [57]	5/23	Home, school	South Africa, Lesotho, Thailand, Cambodia	Diet composition: Most studies (19/23) reported a positive effect. With few exceptions, home garden programs increased fruit and vegetable consumption. Hemoglobin concentration: No statistically significant difference. Vitamin A intake: [MA]: Effect of interventions on serum retinol: SMD = 2.42 (95% CI 1.97 to 2.16, 4 studies, fixed-effects model) Child nutrition status: 1 study found a significant effect on stunting prevalence; 3 studies found positive effects on prevalence of underweight; 2 found a positive effect on wasting.	Meta-analysis provides support that home gardens interventions improve vitamin A intake among children <5. Though results provide little support that agricultural interventions reduce undernutrition, this should not be interpreted as absence of an effect. Lack of significance can be the result of absence of effect or of absence of statistical power, and many studies reviewed included small samples of children.
Mikkelsen *et al*. [58]	3/26	Child care centers, Kindergartens	US, Thailand, Germany	Fruit and vegetable consumption: 6 multicomponent interventions showed a significant increases; 1 found an effect on only fruit consumption after 1 year follow up. Six of the educational only studies showed promising, although non-significant, results. Anthropometric: Educational and multicomponent interventions did not reveal a significant effect on BMI.	Healthy eating interventions in preschools can significantly increase child fruit and vegetable consumption and nutrition-related knowledge if using an educational or multicomponent intervention. Results highlight the scarcity of properly designed interventions with clear indicators and outcomes. Preschool settings could also help decrease child health inequities.
Nekitsing *et al*. [59]	2/30	Child care centers, Kindergartens	US, Thailand	Vegetable intake: [MA] Effect of intervention vs. comparison on vegetable intake: Hedges g = 0.40 (95% CI: 0.31 to 0.50, random effects model, 30 studies, 4017 participants). Funnel plot test suggest that publication bias is present. Subgroup analysis showed intervention effect sizes varied significantly by study design, outcome measure, recipient, strategy, and type of vegetable.	The most successful strategies included taste exposures and reward. Less effective strategies included food services and nutrition education. Meta-regression revealed the more exposure to a vegetable a child receives, the more likely they are to increase intake of that vegetable. Preschoolers may be more amenable to these interventions than older children, therefore early intervention is key.
Ohly *et al*. [31]	2/18	Child care centers	US	Fruit and vegetable intake: 2 studies found significant increases Fruit and vegetable preference: 8/13 interventions reported statistically significant effects Food knowledge and attitudes: Most (7/10) studies found positive effects in the intervention groups. Physical activity: Children in gardening groups reported being less sedentary; spent more time engaged in “moderate” activity compared to control group. Diastolic blood pressure was the only significant effect, which decreased more in the intervention group.	There was little objective evidence for changes in eating habits and physical activity. Stronger evidence supported improvements in knowledge, attitudes and preferences towards fruits and vegetables. Quantitative evidence for health and well-being impacts of school gardening are limited by self-report bias, imperfect measures of fruit and vegetable consumptions, heterogeneity of measurement scales and follow-up time. Students who do not excel in classroom activities were thought to particularly benefit from garden-based interventions.
Savoie-Roskos *et al*. [30]	3/14	Child care centers, school, community	US	Fruit or vegetable consumption: Most articles found improvements after implementation of a gardening intervention. Two studies found that although vegetables consumption at school increased, vegetable consumption at home did not change. Children who received gardening and nutrition education had greater effects when compared to education-only and control groups in 2/3 studies.	Multicomponent garden-based interventions may be effective in increasing fruit and vegetable intake. Garden interventions increased access to fruits and vegetables during the school day. However, children may have limited access at home, resulting in minimal changes over time.
Sisson *et al*. [60]	3/71	Child care centers, preschools, schools	US, Australia	Most studies were successful in promoting change in obesity or obesogenic behaviors and had the intended effect on the target: obesity 48% (14), physical activity 73% (30), diet 87% (39), and screen time 63% (5).	Environment-level interventions had less impact on child health behavior outcomes than those that specifically included child-level interventions. Child care center environment interventions that included technical support facilitated positive changes.

^a^indicates the number of primary studies meeting inclusion criteria out of total primary studies included in this review

#### Effective components of garden-based interventions

Reviews discussed a number of components of garden-based interventions, including hands-on gardening, utilization of produce from garden (e.g., consumption, taste-testing, sale), staff training, nutrition education and cooking components. Amongst reviews, there was consensus that garden-based interventions that aimed to increase healthy eating behaviors should include a nutrition education component, as multi-component interventions may be more effective than garden-only interventions [13, 32]. However, due to limitations in study design and description of interventions, most reviews were unable to report which components of garden-based interventions were most effective for young children, including component effectiveness by setting. Table 4 details a list of garden-based intervention components of included primary studies with our age range of interest by review.

**Table 4:** Components of garden-based interventions in included reviews

	Hands-on gardening	Integration with wider curriculum	Utilized produce from garden	Outdoor garden	Teacher delivered curriculum	Trained staff	Community involvement	Media Promotion	Cooking Component	Nutrition Education	Tasting	Physical Activity	Salad bars
Appleton et al 2016 [49]	X									X		X	X
Beets et al 2009 [50]	X											X	
Berezowitz et al 2015 [27]	X		X	X									
Berti et al 2004	X		X	X									
Bhutta et al 2008 [52]	X		X	X									
Bird et al 2019 [53]	X		X	X									
Davis et al 2015 [54]	X		X		X		X	X	X	X		X	
Hendrie et al 2017 [55]	X	X	X	X	X	X		X	X	X	X		
Hodder et al 2018 [45]	X	X	X	X	X	X							
Langellotto & Gupta 2012	X								X	X		X	X
Massett et al 2012 [58]							X						
Mikkelsen et al 2014	X		X		X		X	X	X	X		X	
Netkitsing et al 2018	X		X		X		X	X	X	X		X	
Ohly et al 2016 [31]	X	X	X	X	X	X					X		
Savoie-Roskos 2017 [30]	X	X	X	X	X	X	X	X	X	X	X		X
Sisson et al 2016 [60]	X	X	X	X	X	X	X	X	X	X	X	X	X

#### Early child health outcomes of garden-based interventions

Garden-based interventions examined numerous health outcomes of garden-based interventions for young children, including nutrition-related, weight status, physical activity, academic performance, mental health, and biological measures. Some studies that examined vegetable intake did so via self-reported measures, whereas others used biological assessments. For example, Faber et al. examined both consumption of yellow and green-leafy vegetables, as well as serum retinol [24]. Garden-based interventions included measures of undernutrition, including stunting, wasting, and underweight [51, 52, 57] but did not report on measures of overweight and obesity for children ages 6 and younger. Biological measures used across garden-based interventions included blood lipids, hemoglobin concentration, and serum retinol. Additional measures included prevalence of night blindness, diarrhea, and respiratory-related infections [51].

Across reviews, there was more evidence for improving nutrition-related outcomes, compared to other outcomes, such as physical activity or academic performance. There was insufficient evidence relating to the effects of garden-based interventions on macronutrient intake. However, there was evidence that garden-based interventions positively affected micronutrient intake, as demonstrated via biological indicators, including serum retinal [52, 57] and hemoglobin concentrations [51]. There was insufficient evidence that garden-based interventions were associated with improvements in anthropometric measures, such as body mass index (BMI) and weight-for-height [53, 58]. There was no evidence that garden-based interventions improved cognitive-related outcomes, including academic performance or mental health in our target age group. Biological measures revealed evidence that garden-based interventions resulted in improvements in prevalence of child anemia [53].

#### Effective settings of garden-based interventions

Across settings, garden-based interventions conducted in the home were consistently associated with improvements in child nutrition status, including wasting [51, 52, 57], stunting [51, 52, 57], and underweight [57]. Moderate evidence across reviews found that garden-based interventions improved fruit and vegetable intake [27, 30, 51, 52, 58, 83], as well as vegetable only intake in the early care and education, school, and home settings [49, 50, 55]. Only 1 garden-based intervention in an early care and education setting examined physical activity as an outcome at follow-up, but found no difference in physical activity levels between the intervention and control groups [80]. As there was only one review that included a community garden-based intervention [55], there was insufficient evidence to examine the effect of gardens within a community setting.

### Recommendations for garden-based interventions

We extracted recommendations for future research and practice related to garden-based interventions across reviews (Table 5). Recommendations for future research included utilization of randomized or quasi-randomized designs with larger sample sizes to establish causal relationships [27, 30, 31, 48, 50, 53–55, 57]. Other recommendations were to improve outcome measures, including more standardized measures to allow for pooling of results for meta-analyses [30, 31, 48, 54–58]. Reviews also recommended examining effects of garden-based interventions on subgroups of children, including children with attention deficit disorders, children from low socioeconomic status groups, and younger children. Practice recommendations included the integration of theory-based [31], age-appropriate garden curricula [27] and age-appropriate evaluation tools [30].

**Table 5:** Summary of research and practice recommendations for garden-based interventions

Research Recommendations	Practice Recommendations
Examine impact on specific subpopulations [30, 31, 49]	Comprehensive school garden interventions [27, 51]
Examine long-term impacts of garden-based interventions [30, 31, 48, 49, 51–56, 58]	Utilization of gardens as a way to improve nutritional outcomes via vegetable provision [31, 49, 52, 55, 58]
Include educational impacts of school-based gardens [27, 31, 54]	Integration of school-gardens into curricular instruction [27]
Assess impact of garden-based interventions on broader community [30, 51, 52, 54]	Develop age-appropriate garden-based curriculum rooted in age-appropriate evaluation tools [30]
Start up and sustainability of school-based gardens [30, 50–52, 54, 55, 58, 59]	Involve parents and staff to achieve buy-in and establish rapport [30, 84]
Enhanced rigor of study design including objective measures, power [27, 30, 31, 48, 50, 53–55, 57]	Multi-disciplinary collaboration, including engagement with local stakeholders and policymakers [51, 53–55]
Cost-effectiveness of garden-based interventions [48, 49, 51–53, 55, 59]	Focus on sustainable behavior change [49, 58]
Enhanced description of intervention methodology, standardized reporting process [27, 31, 55, 56]	Examine instructional quality in delivery of interventions [27]
Increased use of standardized measures of child health outcomes [30, 31, 48, 54–58]	Employ multicomponent interventions, particularly in child care settings [59, 60]
Include qualitative methods [31]	Include taste exposure as part of intervention [59]
Explore mediation effects [54]	
Examine role in neophobia/fussy eating [59]	

## Discussion

This umbrella review provides a comprehensive synthesis of existing systematic review level evidence of the impact of garden-based interventions on health outcomes for children ages 6 years and younger. Of the 16 reviews included in this review, 5 focused exclusively on garden-based interventions [27, 30, 31, 54, 56]. Included reviews varied in quality and included a small number of primary-level garden-based interventions with children within our target age range. Across reviews, nutrition-related health outcomes, such as improving nutritional status and correlates of nutrition status had the most evidence. These included fruit and vegetable intake, mediators of fruit and vegetable intake (e.g., knowledge, willingness to taste, provision), and biological measures (e.g., serum retinol, hemoglobin). There was no review-level evidence that garden-based interventions improved BMI or other anthropometric measures in early childhood.

Across settings, there was the most evidence in support of improved child health outcomes for home gardens, compared to community gardens or early care and education settings. However, few primary level interventions have been conducted in these settings, which may explain the lack of evidence. It may also be that the 3 reviews that included the most relevant primary level studies focused on nutrition-sensitive agriculture in low- to middle-income countries, where interventions typically focus on the home or community setting.

Of included reviews, those from the United States (US) and other high-income countries focused more on obesogenic behaviors (e.g., improving fruit and vegetable intake and knowledge, physical activity, screen time), and reviews of low- to middle-income countries focused on improving undernutrition or nutrition status. This is not a surprising finding, as the US has higher rates of childhood obesity (12.7%), whereas higher rates of undernutrition are more often found in low-income countries [85]. However, with obesity prevalence rising in low- to middle-income countries, the triple burden of malnutrition may prompt changes in intervention approaches toward decreasing the prevalence of obesogenic behaviors and environments [86].

There was insufficient evidence that garden-based interventions were associated with non-nutrition early childhood health outcomes, such as academic performance or physical activity. We found no evidence that garden-based interventions were associated with improvements in developmental milestones or socio-emotional outcomes. For older children, evidence suggests garden-based interventions in the school setting improve academic performance, attendance, and prosocial behaviors [30, 87, 88]. It seems plausible that we would see similar improvements in preschool-aged children in early care and education settings if studies assessed these outcomes via developmentally appropriate measures.

### Review-level evidence gaps

There was evidence that garden-based interventions are linked to improvements in multiple early childhood nutrition outcomes. However, there was little to no evidence on the relationship between garden-based interventions and anthropometric or weight status measures, physical activity, academic performance, and biological measures. A small number of reviews focused exclusively on garden-based interventions; these reviews varied in quality and included less than a dozen garden-based interventions with children in our target age range. Search strategy limitations of included reviews (e.g., inclusion of only a few databases, inadequate search terms, study design) and singular outcomes of interest reduced the number of primary studies included versus existing evidence available. For example, several systematic reviews focused on children, but failed to have critical early childhood search terms necessary to yield relevant studies. In fact, there are several published studies on the effect of garden-based interventions in early childhood that were not included in any included review [26, 89]. There are also several ongoing garden-based interventions with forthcoming results, including a randomized control trial testing the effectiveness of a community-based agricultural intervention [90] and another cluster randomized control trial examining effects of a garden-based early care and education center intervention [91]. These emerging results will, no doubt, contribute to this body of literature.

Although we aimed to examine and report on the components of garden-based interventions that were most effective, we were unable to do so due to limitations in available evidence. First, there was little effort done in the reviews to holistically evaluate effective intervention components. This is probably due, in part, to the array of fields which the reviews were focused and the array of interventions that were included. Therefore, a focus of future research is to determine what type of garden-based intervention components (e.g., cultivation, harvesting, nutrition education, cooking) are most effective. An additional and critical limitation of included reviews is poor quality, when rates via AMSTAR 2. For example, there were just 3 reviews with an a priori protocol specified. Additionally, many reviews did not include, either in the paper or in a supplement, a detailed search strategy or a full list of excluded studies. It is essential that systematic reviews understand the importance of rigorous reporting needed to properly assess existing evidence when undertaking evidence syntheses. Taken together, limitations of previous review create a need for a rigorous systematic review on garden-based interventions and effects on early childhood health outcomes that addresses these crucial limitations of prior systematic reviews.

### Primary study-level evidence gaps

At a primary study level, there is a need for more convincing evidence on the holistic effects of garden-based interventions in child health promotion, particularly among young children, when interventions may have the greatest potential to impact healthy behaviors [59, 92]. Prior systematic reviews reported that most existing studies of garden-based interventions are of poor quality and have numerous limitations. Enhanced rigor of future research in this area, particularly relating to a more robust study design, enhanced measurement of more appropriate outcomes for young children are needed. Additionally, future garden-based interventions should age, developmentally, and culturally tailored. More robust evidence would not only help to delineate any dose-response relationship, but also effectiveness and sustainability of associated benefits.

#### Design of garden-based interventions

Multiple reviews call for age- and setting-specific garden-based interventions, as there is a need to understand what interventions work for which children across settings [31, 45, 59, 93]. There is some evidence that children who struggle in a traditional school environment benefit most from garden-based interventions [94], but the extent to which benefits are enhanced for children among different demographic characteristics remains unknown. Authors of future studies should consider using evidence-based curricula that are tailored to the developmental stages of the intended audience [30, 32]. A few garden-based curricula have been designed for use with young children [14, 95], one of which is “Watch Me Grow”, designed specifically for implementation in early care and education settings [14]. There are many more garden-based curricula available for older children [96–98]. As these curricula have shown positive results, it seems plausible that researchers could easily tailor these curricula and interventions for use during early childhood.

#### Measurement

Measurement issues at a primary-study level, such as lack of standardization across the field [56] and reliance of self-report or parental reports as a proxy for the child’s food preferences and behavior [32, 58], hindered our interpretation of evidence in this review. Multiple reviews called for improved use of validated measures across studies to allow for sophisticated meta-analysis. Standardized measures that future garden-based interventions could use in early childhood include using consistent measuring of fruit and vegetable consumption (e.g., weight vs. volume), validated and reliable scales (e.g., food preference, picky eating, social-emotional health), anthropometrics (BMI, BMI-z score), and the use of biological measures, for which collation is more straightforward [99]. Unfortunately, garden-based interventions rarely used biological measures, which are arguably more resource and time intensive, but may produce more accurate data on health effects. Future studies should incorporate biological sampling methods, as more affordable and feasible assessments become available (e.g., skin carotenoids scans). There is convincing evidence that increasing vegetable consumption is more challenging than increasing fruit consumption, which creates a need for future studies to differentiate between the two when analyzing consumption habits [30]. Additionally, fruit intake is typically higher in young children, which leaves less room for improvement compared to vegetables [12, 30]. Lastly, several reviews mentioned that the use of parental reports as a proxy for the child can be problematic, particularly relating to use of non-validated parental measures or parental measures that are culturally inappropriate. Further, as there are several validated child health measures where the child is the respondent, this would be preferable over parental report, as appropriate.

Beyond standardized child health measures, another important point of consideration for future garden-based interventions in early childhood is to include age-appropriate outcome and evaluation measures. For example, we did not find a single intervention examining developmental milestones or social-emotional outcomes, which would be very interesting to assess in early childhood, where there is an emphases on appropriate social-emotional development. Numerous reliable indicators of child development exist that are both age- and developmentally appropriate (e.g., World Health Organization Motor Milestones, Bayley Scales, Ages and Stages Questionnaire) [100, 101] that could be used in future studies. The Ages and Stages Questionnaire is a short, validated scale that addresses numerous child development components, including motor, cognitive, and social-emotional development) [101] and would be easy to integrate as an outcome.

#### Effective components of garden-based interventions

As mentioned as an evidence gap at the review level, an evidence gap at the primary-study level relates to effective intervention components. Future garden-based interventions could examine intervention effectiveness as a part of the study aim. For example, multiple intervention groups could be formed with a combination of different strategies (e.g., cultivation, harvesting, nutrition education, cooking), enabling comparison of these strategies to the control group. This is a vital area of future research that will enable future studies to be not only more effective, but cost-efficient as well.

### Limitations

There are limitations of this umbrella review. In effort to reduce bias into this review and increase the strength of evidence, we included only systematic reviews that underwent peer-review. In turn, these criteria excluded many potentially relevant reviews that examined garden-based interventions in children [15, 19, 29, 32, 33, 88, 102, 103]. Many of the included systematic reviews were lacking in quality and due to limitations in their search strategy and overall scope. As umbrella reviews inherently rely on the accuracy and quality (e.g., appropriate design, reduced risk of biases, reporting) of included studies, this is a limitation. Incomplete reporting of the AMSTAR 2 elements in the included reviews could have resulted in a poorer quality assessment score. Included reviews often lacked an a priori protocol and a duplicated study screening and data extraction process, which may have introduced bias in their reviews. However, we utilized an a priori protocol and restricted inclusion of reviews to only systematic reviews to reduce potential biases. The date restrictions in our search strategy could have missed recently published reviews not yet indexed in databases.

## Conclusion

We conducted an umbrella review to comprehensively summarize and critically evaluate existing systematic review level evidence of the role of garden-based interventions in early childhood health promotion. However, the full impact of garden-based interventions on early childhood health promotion and associated health outcomes cannot be determined due to limitations within available review- and primary-level evidence. Thus, it is important that this review’s findings not be taken as an absence of an effect, but rather as a product of the limitations of existing evidence. A key finding of this review is that garden-based interventions were most frequently associated with improvements in nutrition-related outcomes, including improvements in nutritional status, fruit and vegetable consumption, micronutrient deficiencies, and enhanced mediators of healthy eating. Until the evidence gaps identified in this umbrella review are addressed, researchers and policymakers should focus on the utility of garden-based interventions as a tool to aid in promoting global early childhood nutrition.

## Supplementary information

**Additional file 1.** Search Strategy. Details search strategy of the review.

**Additional file 2.** List of Excluded Studies. Details list of excluded studies and reason for exclusion at the full-text review level.

**Additional file 3.** Overlap Matrix. Details the matrix used to calculate overlap at the primary study level.

**Additional file 4.** PRISMA Checklist. PRISMA Checklist for Systematic Reviews.

## Data Availability

The data used and analyzed during the current study are available from the corresponding author upon reasonable request.

## Abbreviations

JBI: Joanna Briggs Institute
PRISMA: Preferred Reporting Items for Systematic Reviews and Meta-Analyses Protocols
BMI: Body Mass Index
AMSTAR: A Measurement Tool to Assess Systematic Reviews
