# Supplementary material for: Garden-based interventions and early childhood health: an umbrella review
Source: Int J Behav Nutr Phys Act. 2020 Sep 22;17:121. doi: 10.1186/s12966-020-01023-5 (PMC7509938; doi:10.1186/s12966-020-01023-5)
Supplement: Supplementary file 1 — Additional file 1. Search Strategy. Details search strategy of the review. [file 12966_2020_1023_MOESM1_ESM.docx]

**Additional File 1: Search Strategy**

Lines 1 to 5 (in black) detail the final search strategy that will be used for the umbrella review. The remaining searches in **blue**, detail the full pilot search strategy. The pilot search strategy was run on the 9^th^ of January 2019 using a mixture of controlled vocabulary and key words.

| 1 | TI (child* or childhood or kids or ‘young children’) OR TI (preschool or kindergarten or ‘early childhood education’ or ‘day care’ or ‘child care’) OR TI (early care or ECE) OR AB (child* or childhood or kids or ‘young children’) OR AB (preschool or kindergarten or ‘early childhood education’ or ‘day care’ or ‘child care’) OR AB (‘early care’ or ECE) OR TI (Head Start) OR AB (Head start) |
| --- | --- |
| 2 | TI (garden* or horticulture or horticulture therapy) OR TI (farm to preschool) OR TI (farming or agriculture) OR TI (fruit or vegetable) OR AB (garden* or horticulture or horticulture therapy) OR AB (farm to preschool) OR AB (farming or agriculture) OR AB (fruit or vegetable) |
| 3 | TI (systematic review or review or meta-analysis or narrative review) OR AB (systematic review or review or meta-analysis or narrative review) |
| 4 | 1 AND 2 |
| 5 | 3 AND 4 |
| 6 | Microfarms or grow or food science or crops or allotment or cultivate |
| 7 | Creche or Camp or center-based childcare |
| 8 | 2 AND 6 |
| 9 | 1 AND 7 |
| 10 | 3 AND 8 AND 9 |
| 11 | TI (pediatric or paediatric) OR AB (pediatric or paediatric) |
| 12 | TI (gardening-based or farm-to-preschool) or AB (gardening-based or farm-to-preschool) |
| 13 | 1 AND 11 |
| 14 | 2 AND 12 |
| 15 | TI (literature) |
| 16 | 3 AND 15 |
| 17 | 13 AND 14 AND 16 |
