# Supplementary material for: Garden-based interventions and early childhood health: an umbrella review
Source: Int J Behav Nutr Phys Act. 2020 Sep 22;17:121. doi: 10.1186/s12966-020-01023-5 (PMC7509938; doi:10.1186/s12966-020-01023-5)
Supplement: Supplementary file 2 — Additional file 2. List of Excluded Studies. Details list of excluded studies and reason for exclusion at the full-text review level. [file 12966_2020_1023_MOESM2_ESM.docx]

**Additional File 2: List of excluded studies and reason for exclusion**

| Study | Title | Reason for Exclusion |
| --- | --- | --- |
| Aguilar 2008 | Growing environmental stewards: The overall effect of a school gardening program on environmental attitudes and environmental locus of control of different demographic groups of elementary school children | Not a systematic review |
| Agustina 2012 | Community gardens: Space for interactions and adaptations | Not a systematic review |
| Ahuja 2016 | Importance of nutrition education in treating and preventing obesity in minority populations in the United States | No garden-based intervention |
| Alaimo 2016 | Harvesting Health in the Garden | Not a systematic review |
| Al-Delaimy 2017 | Community gardens as environmental health interventions: Benefits versus potential risks | Not a systematic review |
| Aloia 2016 | Pertinence of the recent school-based nutrition interventions targeting fruit and vegetable consumption in the United States: a systematic review | Does not include children under 6 years of age |
| Ambrosini 2014 | Childhood dietary patterns and later obesity: A review of the evidence | Not peer reviewed |
| Ammerman 2002 | The efficacy of behavioral interventions to modify dietary fat and fruit and vegetable intake: a review of the evidence | No garden-based intervention |
| Amoroso 2018 | Post-2015 Agenda and sustainable development goals: Where are we now? Global opportunities to address malnutrition in all its forms, including hidden hunger | Not a systematic review |
| Ananthapavan 2019 | Cost-effectiveness of community-based childhood obesity prevention interventions in Australia | No child health or well-being outcome |
| Appleton 2017 | Increasing Vegetable Intakes: An Updated Systematic Review of Published Interventions | Not peer reviewed |
| Ardoin 2018 | Environmental education and K-12 student outcomes: A review and analysis of research | No garden-based intervention |
| Artz 2017 | Green Care: A Review of the Benefits and Potential of Animal-Assisted Care Farming Globally and in Rural America | No garden-based intervention |
| Baker 2004 | Tending cultural landscapes and food citizenship in Toronto's community gardens | Not a systematic review |
| Bergman 2016 | Using community-based participatory research to create sustainable healthy school environments and promote positive behavior changes in school-aged children | No garden-based intervention |
| Berlin 2013 | The Role of Social Cognitive Theory in Farm-to-School-Related Activities: Implications for Child Nutrition | Not a systematic review |
| Bernardon 2014 | School Gardens in the Distrito Federal, Brazil | Not a systematic review |
| Bice 2018 | Community Gardens: Interactions between Communities, Schools, and Impact on Students | Not a systematic review |
| Birch 2009 | Preventing childhood obesity: What works? | Not a systematic review |
| Black 2017 | How effective are family-based and institutional nutrition interventions in improving children's diet and health? A systematic review | Does not include children under 6 years of age |
| Blair 2009 | The child in the garden: An evaluative review of the benefits of school gardening | Does not include children under 6 years of age |
| Bourke 2014 | Are dietary interventions effective at increasing fruit and vegetable consumption among overweight children? A systematic review | No garden-based intervention |
| Branscum 2012 | After-school based obesity prevention interventions: a comprehensive review of the literature | Does not include children under 6 years of age |
| Braun 2006 | A global survey and review of farmer field school experiences | Not peer reviewed |
| Brennan 2011 | Accelerating evidence reviews and broadening evidence standards to identify effective, promising, and emerging policy and environmental strategies for prevention of childhood obesity | No garden-based intervention |
| Brown 2016 | A Systematised Review of Primary School Whole Class Child Obesity Interventions: Effectiveness, Characteristics, and Strategies | No garden-based intervention |
| Browning 2019 | School green space and its impact on academic performance: a systematic literature review | No garden-based intervention |
| Buchsbaum 2016 | Review of the micronutrient impact of multi-sectoral programmes focusing on nutrition | Not a systematic review |
| Bundara 2013 | Addressing childhood undernutrition in Tanzania: challenges and opportunities | Not a systematic review |
| Burchett 2003 | Increasing fruit and vegetable consumption among British primary schoolchildren: A review | No garden-based intervention |
| Campbell 2007 | Strategies which aim to positively impact on weight, physical activity, diet and sedentary behaviours in children from zero to five years. A systematic review of the literature | No garden-based intervention |
| Carlsson 2008 | New approaches to the health promoting school: participation in sustainable food systems | Not a systematic review |
| Casanovas 2013 | Multi-sectoral interventions for healthy growth | No garden-based intervention |
| Chaffee 2014 | Early life factors among the many influences of child fruit and vegetable consumption | Not a systematic review |
| Chaifetz 2015 | Implementation of good agricultural practices (GAPs) in school and community gardens | Not a systematic review |
| Chamhuri 2014 | Household food insecurity, nutritional outcome and coping strategies: evidence from Malaysia | No garden-based intervention |
| Chan 2017 | Association between dietary intake and 'school-valued' outcomes: a scoping review | No garden-based intervention |
| Chaufan 2015 | Advancing family health through the Garden of Eatinâ€™: On-site food gardens in early childhood education | Not a systematic review |
| Ciliska 2000 | Effectiveness of community-based interventions to increase fruit and vegetable consumption | No garden-based intervention |
| Clatworthy 2013 | Gardening as a mental health intervention: a review | Does not include children under 6 years of age |
| Colley 2018 | The Impact of Canadian School Food Programs on Childrenâ€™s Nutrition and Health: A Systematic Review | No garden-based intervention |
| Collins 2013 | Effectiveness of parent-centred interventions for the prevention and treatment of childhood overweight and obesity in community settings: A systematic review | No garden-based intervention |
| Cook 2015 | Association between home availability and vegetable consumption in youth: a review | Does not include children under 6 years of age |
| Corrigan 2011 | Growing what you eat: Developing community gardens in Baltimore, Maryland | Not a systematic review |
| Dart 2010 | Community gardening and education outreach expand students dietetics practice skills | Not a systematic review |
| Davies 2015 | Learning in cultivated gardens and other outdoor landscapes | Not a systematic review |
| Davis 2017 | Examining the Benefits and Barriers of Instructional Gardening Programs to Increase Fruit and Vegetable Intake among Preschool-Age Children | Not a systematic review |
| DeCosta 2017 | Changing children's eating behaviour - A review of experimental research | Does not include children under 6 years of age |
| Delgado-Noguera 2011 | Primary school interventions to promote fruit and vegetable consumption: a systematic review and meta-analysis | Not a systematic review |
| DeMattia 2008 | Childhood obesity prevention: Successful community-based efforts | Not a systematic review |
| Dennis 2017 | Evaluating the relative influence on population health of domestic gardens and green space along a rural-urban gradient | Not a systematic review |
| deSa 2008 | Will European agricultural policy for school fruit and vegetables improve public health? A review of school fruit and vegetable programmes | Does not include children under 6 years of age |
| Diep 2014 | Influence of behavioral theory on fruit and vegetable intervention effectiveness among children: a meta-analysis | Does not include children under 6 years of age |
| Dillon 2003 | Improving the understanding of food, farming and land management amongst school-age children: A literature review | Not peer reviewed |
| Dillon 2006 | The value of outdoor learning: evidence from research in the UK and elsewhere | No garden-based intervention |
| Doak 2006 | The prevention of overweight and obesity in children and adolescents: a review of interventions and programmes | No garden-based intervention |
| Doerfler 2011 | Using school gardens as a vehicle for health promotion for elementary school youth: a review of the literature | Not peer reviewed |
| Domenghini 2011 | Physical activity and curriculum development of an after-school gardening program for youth health | Not a systematic review |
| Dovey 2008 | Food neophobia and 'picky/fussy' eating in children: A review | No garden-based intervention |
| Downes 2016 | Evidence for Using Farm Care Practices to Improve Attachment Outcomes in Foster Children: A Systematic Review | No garden-based intervention |
| Draper 2010 | Review and analysis of the benefits, purposes, and motivations associated with community gardening in the United States | Not a systematic review |
| Dudley 2015 | Teaching approaches and strategies that promote healthy eating in primary school children: a systematic review and meta-analysis | Does not include children under 6 years of age |
| Dunton 2009 | Physical environmental correlates of childhood obesity: a systematic review | No garden-based intervention |
| Dwyer 2002 | Evaluating school-based interventions using the Healthy Eating Index | Not a systematic review |
| Dyment 2007 | Grounds for movement: green school grounds as sites for promoting physical activity | Not a systematic review |
| Eigenbrod 2015 | Urban vegetable for food security in cities. A review | No child health or well-being outcome |
| Evans 2010 | 068â€…Systematic review and meta-analysis of school-based interventions to improve fruit and vegetable intake | Not a systematic review |
| Evans 2012 | Systematic review and meta-analysis of school-based interventions to improve daily fruit and vegetable intake in children aged 5 to 12 y | No garden-based intervention |
| Fanzo 2011 | A review of global progress toward the Millennium Development Goal 1 Hunger Target | No child health or well-being outcome |
| Feenstra 2012 | The evolution of the school food and farm to school movement in the United States: connecting childhood health, farms, and communities | No child health or well-being outcome |
| Ferris 2001 | People, land and sustainability: Community gardens and the social dimension of sustainable development | No garden-based intervention |
| Fiorella 2016 | Agricultural interventions for improved nutrition: A review of livelihood dimensions | Not a systematic review |
| Fischer 2019 | Biodiverse edible schools: Linking healthy food, school gardens and local urban biodiversity | Not a systematic review |
| French 2003 | Environmental interventions to promote vegetable and fruit consumption among youth in school settings | No garden-based intervention |
| Ganann 2012 | Community-based interventions for enhancing access to or consumption of fruit and vegetables among five to 18-year olds: A scoping review | Not a systematic review |
| Ganann 2014 | Enhancing nutritional environments through access to fruit and vegetables in schools and homes among children and youth: a systematic review | Does not include children under 6 years of age |
| GardnerBurt 2016 | A complete history of the social, health, and political context of the school gardening movement in the United States: 1840â€“2014 | Does not include children under 6 years of age |
| Geller 2009 | Longitudinal and cross-sectional influences on youth fruit and vegetable consumption | No garden-based intervention |
| Genter 2015 | The contribution of allotment gardening to health and wellbeing: A systematic review of the literature | Does not include children under 6 years of age |
| Genuneit 2012 | Exposure to farming environments in childhood and asthma and wheeze in rural populations: a systematic review with meta-analysis | No garden-based intervention |
| Ghosh 2010 | Sustainability potential of suburban gardens: review and new directions | No child health or well-being outcome |
| Graziose 2018 | Factors Related to Fruit and Vegetable Consumption at Lunch Among Elementary Students: A Scoping Review | Does not include children under 6 years of age |
| Guitart 2012 | Past results and future directions in urban community gardens research | No child health or well-being outcome |
| Hamel 2013 | Computer- and web-based interventions to promote healthy eating among children and adolescents: a systematic review | No garden-based intervention |
| Haselow 2016 | Evidence-based evolution of an integrated nutrition-focused agriculture approach to address the underlying determinants of stunting | Not a systematic review |
| Hawkes 2015 | Smart food policies for obesity prevention | No garden-based intervention |
| Hayes-Conroy 2010 | School gardens and 'actually existing' neoliberalism | No child health or well-being outcome |
| Henderson 2004 | Whole-school approaches to sustainability: An international review of sustainable school programs | Not peer reviewed |
| Hendrie 2012 | Combined home and school obesity prevention interventions for children: What behavior change strategies and intervention characteristics are associated with effectiveness? | No garden-based intervention |
| Hendrie 2013 | Improving children's dairy food and calcium intake: Can intervention work? A systematic review of the literature | No garden-based intervention |
| Hersch 2014 | The impact of cooking classes on food-related preferences, attitudes, and behaviors of school-aged children: a systematic review of the evidence, 2003-2014 | Does not include children under 6 years of age |
| Hesketh 2010 | Interventions to prevent obesity in 0-5 year olds: An updated systematic review of the literature | No garden-based intervention |
| Hill 2004 | Family and community practices that promote child survival, growth and development: a review of the evidence | Not peer reviewed |
| Hoffman 2017 | Farm to Preschool: The State of the Research Literature and a Snapshot of National Practice | Not a systematic review |
| Holley 2017 | A Systematic Review of Methods for Increasing Vegetable Consumption in Early Childhood | No garden-based intervention |
| Holley 2019 | A Systematic Review of the Evaluation of Interventions to Tackle Childrenâ€™s Food Insecurity | Does not include children under 6 years of age |
| Howerton 2007 | School-based Nutrition Programs Produced a Moderate Increase in Fruit and Vegetable Consumption: Meta and Pooling Analyses from 7 Studies | No garden-based intervention |
| Huelskamp 2018 | Enhancing the Health of School Garden Programs and Youth: A Systematic Review | No child health or well-being outcome |
| Hussein 2009 | Therapeutic Intervention: Using Sensory Gardens to Enhance the Quality of Life for Children with Special Needs | Not a systematic review |
| Hussein 2014 | Sensory gardens | Not a systematic review |
| Iaquinta 2010 | Urban agriculture: a comparative review of allotment and community gardens | Not a systematic review |
| Jiang 2014 | Therapeutic landscapes and healing gardens: A review of Chinese literature in relation to the studies in western countries | Does not include children under 6 years of age |
| Jones 2012 | Improving health, building community: Exploring the asset building potential of community gardens | Does not include children under 6 years of age |
| Joshi 2008 | Do farm-to-school programs make a difference? findings and future research needs | Not a systematic review |
| Kabisch 2017 | The health benefits of nature-based solutions to urbanization challenges for children and the elderly - A systematic review | No garden-based intervention |
| Kaczynski 2007 | Environmental correlates of physical activity: a review of evidence about parks and recreation | No garden-based intervention |
| Keatinge 2012 | Vegetable gardens and their impact on the attainment of the Millennium Development Goals | No garden-based intervention |
| Kiraly 2017 | Applying Ecological Frameworks in Obesity Intervention Studies in Hispanic/Latino Youth: A Systematic Review | No garden-based intervention |
| Knai 2006 | Getting children to eat more fruit and vegetables: a systematic review | No garden-based intervention |
| Kobes 2018 | Interventions aimed at preventing and reducing overweight/obesity among children and adolescents: a metaâ€synthesis | No garden-based intervention |
| Kropski 2008 | School-based obesity prevention programs: An evidence-based review | No garden-based intervention |
| Kunpeuk 2019 | The impact of gardening on nutrition and physical health outcomes: a systematic review and meta-analysis | Does not include children under 6 years of age |
| Kuzevanov 2006 | Botanic gardens resources: tangible and intangible aspects of linking biodiversity and human well-being | Not a systematic review |
| Lachowycz 2011 | Greenspace and obesity: a systematic review of the evidence | No garden-based intervention |
| Langford 2014 | The WHO Health Promoting School framework for improving the health and well-being of students and their academic achievement | No garden-based intervention |
| Langford 2015 | The World Health Organization's Health Promoting Schools framework: a Cochrane systematic review and meta-analysis | No garden-based intervention |
| Langford 2015 | Obesity prevention and the Health promoting Schools framework: essential components and barriers to success | No garden-based intervention |
| Larson 2011 | What role can child-care settings play in obesity prevention? A review of the evidence and call for research efforts | No garden-based intervention |
| Ling 2016 | Interventions to prevent and manage overweight or obesity in preschool children: A systematic review | No garden-based intervention |
| Madden 2018 | Change and Maintaining Change in School Cafeterias: Economic and Behavioral-Economic Approaches to Increasing Fruit and Vegetable Consumption | Not a systematic review |
| Matson-Koffman 2005 | A site-specific literature review of policy and environmental interventions that promote physical activity and nutrition for cardiovascular health: What works? | Does not include children under 6 years of age |
| McAlister 2013 | The role of botanic gardens in health and well being | Not a systematic review |
| McCormack 2010 | Review of the nutritional implications of farmers' markets and community gardens: a call for evaluation and research efforts | No garden-based intervention |
| McCormick 2017 | Does Access to Green Space Impact the Mental Well-being of Children: A Systematic Review | No garden-based intervention |
| McCrorie 2014 | Combining GPS, GIS, and accelerometry to explore the physical activity and environment relationship in children and young people - a review | No garden-based intervention |
| McNab 2012 | Effective leadership to alter school food environments and improve public health | Not a systematic review |
| Merrey 2014 | Review paper on 'Garden Kits' in Africa: lessons learned and the potential of improved water management | Not a systematic review |
| Micha 2018 | Effectiveness of school food environment policies on children's dietary behaviors: A systematic review and meta-analysis | No garden-based intervention |
| Militello 2018 | A review of systematic reviews targeting the prevention and treatment of overweight and obesity in adolescent populations | Not a systematic review |
| Miller 2007 | The seeds of learning: Young children develop important skills through their gardening activities at a midwestern early education program | Not a systematic review |
| Miyoshi 2012 | School-based "Shokuiku" program in Japan: Application to nutrition education in Asian countries | Not a systematic review |
| Morris 2000 | School-based gardens can teach kids healthier eating habits | Does not include children under 6 years of age |
| Muckelbauer 2011 | Childhood overweight and obesity: Introduction into epidemiology and prevention strategies | Not a systematic review |
| Muehlhoff 2011 | Introducing vegetables into the India Mid-day Meal (MDM) programme: the potential for dietary change | Not a systematic review |
| Muehlhoff 2017 | Linking agriculture and nutrition education to improve infant and young child feeding: lessons for future programmes | Not a systematic review |
| Murimi 2018 | Factors that contribute to effective nutrition education interventions in children: a systematic review | Does not include children under 6 years of age |
| Mushi-Brunt 2009 | Fruit and vegetable intake and weight status among pre-adolescent children: An ecological perspective | Not peer reviewed |
| Muzaffar 2018 | Narrative Review of Culinary Interventions with Children in Schools to Promote Healthy Eating: Directions for Future Research and Practice | Does not include children under 6 years of age |
| Nekitsing 2018 | Developing Healthy Food Preferences in Preschool Children Through Taste Exposure, Sensory Learning, and Nutrition Education | No garden-based intervention |
| Nga 2019 | School education and childhood obesity: A systemic review | No garden-based intervention |
| Nishii 2012 | The therapeutic benefits of gardening: Cultivating health through interaction with nature | Not peer reviewed |
| Noy 2018 | New frontiers in community initiatives to increase vegetable consumption | Not a systematic review |
| Okvat 2011 | Community gardening: A parsimonious path to individual, community, and environmental resilience | Not a systematic review |
| Oldroyd 2008 | The effectiveness of nutrition interventions on dietary outcomes by relative social disadvantage: a systematic review | No garden-based intervention |
| Olstad 2017 | Can targeted policies reduce obesity and improve obesity-related behaviours in socioeconomically disadvantaged populations? A systematic review | No garden-based intervention |
| Organic 2014 | The benefits of gardening and food growing for health and wellbeing | Not a systematic review |
| Orsini 2013 | Urban agriculture in the developing world: a review | No child health or well-being outcome |
| Oxenham 2010 | School Gardens as a Strategy for Increasing Fruit and Vegetable Consumption | Not a systematic review |
| Ozer 2006 | The effects of school gardens on students and schools: Conceptualization and considerations for maximizing healthy development | Does not include children under 6 years of age |
| Ozer 2007 | The effects of school gardens on students and schools: Conceptualization and considerations for maximizing healthy development | Does not include children under 6 years of age |
| PÃ©rez-Morales 2009 | Randomized controlled school based interventions to prevent childhood obesity: Systematic review from 2006 to 2009 | No garden-based intervention |
| Pandey 2016 | Impact of agricultural interventions on the nutritional status in South Asia: A review | Not a systematic review |
| Park 2013 | The metabolic costs of gardening tasks in children | Not a systematic review |
| Park 2014 | Comparison of the metabolic costs of gardening and common physical activities in children | Not a systematic review |
| Park 2016 | Horticultural activity interventions and outcomes: a review | Not a systematic review |
| Parmer 2009 | School gardens: an experiential learning approach for a nutrition education program to increase fruit and vegetable knowledge, preference, and consumption among second-grade students | Not a systematic review |
| Pasha 2013 | Barriers to garden visitation in children's hospitals | Not a systematic review |
| Pate 2009 | After-school interventions to increase physical activity among youth | Does not include children under 6 years of age |
| Poulsen 2015 | A systematic review of urban agriculture and food security impacts in low-income countries | No garden-based intervention |
| Pradhan 2016 | Nutrition interventions for children aged less than 5 years following natural disasters: a systematic review | No garden-based intervention |
| Rasmussen 2006 | Determinants of fruit and vegetable consumption among children and adolescents: a review of the literature. Part I: Quantitative studies | No garden-based intervention |
| Raveendra 2014 | Sensory gardens for disabled: a review | No child health or well-being outcome |
| Robinson-O'Brien 2009 | Impact of garden-based youth nutrition intervention programs: a review | Not a systematic review |
| Roncarolo 2016 | Short-term effects of traditional and alternative community interventions to address food insecurity | Not a systematic review |
| Rosenkranz 2008 | Impacting the home environment toward the prevention of childhood obesity | Not peer reviewed |
| Rossin-Slater 2015 | Promoting health in early childhood | No garden-based intervention |
| Ruel 2004 | Features of urban food and nutrition security and considerations for successful urban programming | Not a systematic review |
| Ruel 2013 | Nutrition-sensitive interventions and programmes: how can they help to accelerate progress in improving maternal and child nutrition? | Not a systematic review |
| Rush 2014 | Systematic review of school and community-based fruit and vegetable interventions for minority children | Does not include children under 6 years of age |
| Ryan-Krause 2018 | Gardening: A Path to Development And Health | No garden-based intervention |
| SÃ¶derback 2004 | Horticultural therapy: the â€˜healing gardenâ€™and gardening in rehabilitation measures at Danderyd Hospital Rehabilitation Clinic, Sweden | Not a systematic review |
| Sahay 2006 | Effective Components for Nutrition Interventions: A Review and Application of the Literature | No garden-based intervention |
| SantillanaMarÃ­n 2013 | [Programs aimed to increase the nutritional content of lunch packs; systematic review] | No garden-based intervention |
| Scherr 2013 | Integrating local agriculture into nutrition programs can benefit children's health | Not a systematic review |
| Sempik 2003 | Social and therapeutic horticulture: evidence and messages from research | Not peer reviewed |
| Sharma 2007 | International schoolâ€based interventions for preventing obesity in children | No garden-based intervention |
| Sharma 2011 | Dietary education in school-based childhood obesity prevention programs | Does not include children under 6 years of age |
| Sharma 2015 | Multidisciplinary approaches to address food insecurity and nutrition among youth and their families | Not a systematic review |
| Sherry 2005 | Food behaviors and other strategies to prevent and treat pediatric overweight | No garden-based intervention |
| Shiu 2012 | Nurturing healthy dietary habits among children and youth in Singapore | No garden-based intervention |
| Showell 2013 | A systematic review of home-based childhood obesity prevention studies | No garden-based intervention |
| Silveira 2011 | Effectiveness of school-based nutrition education interventions to prevent and reduce excessive weight gain in children and adolescents: a systematic review | No garden-based intervention |
| Simon 2012 | Food stamps grow urban gardens | Not a systematic review |
| Somerset 2009 | Variations in prevalence and conduct of school food gardens in tropical and subtropical regions of north-eastern Australia | Not a systematic review |
| Specchia 2018 | Highly-integrated programs for the prevention of obesity and overweight in children and adolescents: results from a systematic review and meta-analysis | No garden-based intervention |
| Spurrier 2009 | Preventing childhood obesity: The effects of nutritional education on increasing fruit and vegetable consumption in preschoolers | Not peer reviewed |
| Stein 2008 | Community gardens for health promotion and disease prevention | Not a systematic review |
| Story 2009 | Schools and obesity prevention: creating school environments and policies to promote healthy eating and physical activity | Not a systematic review |
| Study | Title | Reason for Exclusion |
| Swartz 2018 | Produce Rx Programs for Diet-Based Chronic Disease Prevention | No garden-based intervention |
| Swinburn 2004 | Diet, nutrition and the prevention of excess weight gain and obesity | No garden-based intervention |
| Tampoukou 2015 | Teachersâ€™ Perceptions Î¿n the Use of Botanic Gardens as a Means of Environmental Education in Schools and the Enhancement of School Student Benefits from Botanic Garden Visits | Not a systematic review |
| Tan 2009 | â€œCommunity in Bloomâ€: local participation of community gardens in urban Singapore | Not a systematic review |
| Taylor 1995 | Contextualising the Curriculum in Rural Primary Schools: The Role of Agriculture | Not a systematic review |
| Taylor 1997 | Contextualising Teaching and Learning in Rural Primary Schools: Using Agricultural Experience. Volume 1 [and] Volume 2. Education Research | Not a systematic review |
| Taylor 2013 | Farm to School as a strategy to increase children's fruit and vegetable consumption in the United States: Research and recommendations | Not a systematic review |
| Taylor 2014 | Urban home food gardens in the Global North: research traditions and future directions | Not a systematic review |
| Taylor 2015 | Increasing primary school childrenâ€™s fruit and vegetable consumption: A review of the Food Dudes programme | No garden-based intervention |
| Temple 2014 | A systematic review of interventions to promote physical activity in the preschool setting | No garden-based intervention |
| Thomson 2011 | A systematic review of behavioral interventions to promote intake of fruit and vegetables | Does not include children under 6 years of age |
| Tirivayi 2016 | The interaction between social protection and agriculture: A review of evidence | No garden-based intervention |
| Touyz 2018 | Parent-targeted home-based interventions for increasing fruit and vegetable intake in children: a systematic review and meta-analysis | No garden-based intervention |
| Turner 2016 | Increasing prevalence of US elementary school gardens, but disparities reduce opportunities for disadvantaged students | Not a systematic review |
| Vallianatos 2004 | Farm-to-school: Strategies for urban health, combating sprawl, and establishing a community food systems approach | No child health or well-being outcome |
| Vesilind 1998 | Gardens or graveyards: Science education reform and school culture | Not a systematic review |
| VonSchirnding 2002 | Health and sustainable development: Can we rise to the challenge? | Not a systematic review |
| Wadhera 2015 | Teaching children to like and eat vegetables | No garden-based intervention |
| Wake 2008 | â€˜In the best interests of the childâ€™: Juggling the geography of children's gardens (between adult agendas and children's needs) | Not a systematic review |
| Wakefield 2007 | Growing urban health: community gardening in South-East Toronto | Not a systematic review |
| Wang 2013 | The implementation and effectiveness of school-based nutrition promotion programmes using a health-promoting schools approach: a systematic review | Does not include children under 6 years of age |
| Wang 2015 | What childhood obesity prevention programmes work? A systematic review and meta-analysis | No garden-based intervention |
| Wansink 2014 | Smarter lunchrooms - does changing environments really give more nutritional bang for the buck? | No garden-based intervention |
| Waters 2011 | Interventions for preventing obesity in children | Not a systematic review |
| Weare 2013 | Promoting mental, emotional and social health: A whole school approach | Not a systematic review |
| Webb 2014 | Impacts of agriculture on nutrition: nature of the evidence and research gaps | Not a systematic review |
| Weinstein 2014 | Museums, zoos, and gardens: How formal-informal partnerships can impact urban studentsâ€™ performance in science | Not a systematic review |
| Wells 2015 | The Infusion of Inquiry-Based Learning into School-Based Agricultural Education: A Review of Literature | No garden-based intervention |
| West* 2004 | School effects on pupils' health behaviours: evidence in support of the health promoting school | Not a systematic review |
| Whitehouse 2001 | Evaluating a children's hospital garden environment: Utilization and consumer satisfaction | Not a systematic review |
| Wielgosz 2014 | Agro-ecology, household economics and malaria in Uganda: Empirical correlations between agricultural and health outcomes | Not a systematic review |
| Wilding 2019 | Maternal and earlyâ€life areaâ€level characteristics and childhood adiposity: A systematic review | No garden-based intervention |
| Williams 2013 | Impact of garden-based learning on academic outcomes in schools: Synthesis of research between 1990 and 2010 | Not a systematic review |
| Williams 2014 | A systematic review of the influence of the retail food environment around schools on obesity-related outcomes | No garden-based intervention |
| Williams 2015 | Strategies for enhancing the implementation of school-based policies or practices targeting risk factors for chronic disease | Not a systematic review |
| Wolfenden 2017 | Strategies for enhancing the implementation of school-based policies or practices targeting risk factors for chronic disease | Does not include children under 6 years of age |
| Wolsey 2014 | School gardens: Situating students within a global context | Not a systematic review |
| Yost 2009 | Benefits of gardening for children | Not peer reviewed |
| Zenzen 2009 | Integrative Review of School-based Childhood Obesity Prevention Programs | No garden-based intervention |
| Zhou 2014 | Childhood obesity prevention interventions in childcare settings: Systematic review of randomized and nonrandomized controlled trials | No garden-based intervention |
