# Supplementary material for: Garden-based interventions and early childhood health: an umbrella review
Source: Int J Behav Nutr Phys Act. 2020 Sep 22;17:121. doi: 10.1186/s12966-020-01023-5 (PMC7509938; doi:10.1186/s12966-020-01023-5)
Supplement: Supplementary file 3 — Additional file 3. Overlap Matrix. Details the matrix used to calculate overlap at the primary study level. [file 12966_2020_1023_MOESM3_ESM.docx]

**Additional File 3: Overlap Matrix**

|  | Appleton et al 2016 | Beets et al 2009 | Berezowitz et al 2015 | Berti et al 2004 | Bhutta et al 2008 | Bird et al 2019 | Davis et al 2015 | Hendrie et al 2017 | Hodder et al 2018 | Langellotto & Gupta 2012 | Masset et al 2012 | Mikkelsen et al 2014 | Netkitsing et al 2018 | Ohly et al 2016 | Savoie-Roskos 2017 | Sisson et al 2016 |
| --- | --- | --- | --- | --- | --- | --- | --- | --- | --- | --- | --- | --- | --- | --- | --- | --- |
| Faber et al 2002 | **X** |  |  |  | **X** |  |  |  |  |  | **X** |  |  |  |  |  |
| Wright & Rowell et al. 2010 | **X** |  |  |  |  |  | **X** |  |  | **X** |  |  |  |  |  |  |
| Hermann et al 2006 |  | **X** |  |  |  |  | **X** |  |  | **X** |  |  |  |  |  |  |
| Meinen et al. 2012 |  |  | **X** |  |  |  |  |  |  |  |  |  |  |  | **X** |  |
| Marsh et al. 1998 |  |  |  | **X** |  |  |  |  |  |  |  |  |  | **X** |  |  |
| English et al. 1997 |  |  |  | **X** | **X** |  |  |  |  |  |  |  |  |  |  |  |
| Phillips et al. 1996 |  |  |  | **X** | **X** |  |  |  |  |  |  |  |  |  |  |  |
| Smitasiri et al. 1999 |  |  |  | **X** | **X** |  |  |  |  |  |  |  |  |  |  |  |
| Brun et al. 1991 |  |  |  | **X** |  |  |  |  |  |  |  |  |  |  |  |  |
| Chang et al. 1994 |  |  |  |  | **X** |  |  |  |  |  |  |  |  |  |  |  |
| Malekafzali et al. 2000 |  |  |  |  | **X** |  |  |  |  |  |  |  |  |  |  |  |
| Khamjoung et al. 2000 |  |  |  |  | **X** |  |  |  |  |  |  |  |  |  |  |  |
| Birdi et al. 2015 |  |  |  |  |  | **X** |  |  |  |  |  |  |  |  |  |  |
| Osei et al 2017 |  |  |  |  |  | **X** |  |  |  |  |  |  |  |  |  |  |
| Castro et al 2013 |  |  |  |  |  |  |  | **X** |  |  |  |  |  |  | **X** |  |
| Namenek-Brouwer et al.2013 |  |  |  |  |  |  |  | **X** | **X** |  |  | **X** | **X** | **X** | **X** | **X** |
| Laurie et al. 2008 |  |  |  |  |  |  |  |  |  |  | **X** |  |  |  |  |  |
| Makhotia et al. 2004 |  |  |  |  |  |  |  |  |  |  | **X** |  |  |  |  |  |
| Schipani et al 2002 |  |  |  |  |  |  |  |  |  |  | **X** |  |  |  |  |  |
| Olney et al. 2009 |  |  |  |  |  |  |  |  |  |  | **X** |  |  |  |  |  |
| Sirikulchayanonta et al. 2010 |  |  |  |  |  |  |  |  |  |  |  | **X** | **X** |  |  |  |
| De Bock et al. 2011 |  |  |  |  |  |  |  |  |  |  |  | **X** |  |  |  |  |
| Adams et al. 2009 |  |  |  |  |  |  |  |  |  |  |  |  |  |  |  | **X** |
| Farfan-Ramirez et al. 2011 |  |  |  |  |  |  |  |  |  |  |  |  |  |  |  | **X** |
